# Downregulation of TCF19 and ATAD2 causes endothelial cell cycle arrest at the transition from cardiac hypertrophy to heart failure

**DOI:** 10.1007/s00395-025-01139-4

**Published:** 2025-09-17

**Authors:** Elias Erny, Christoph Koentges, Debanjan Mukherjee, Luisa Wirth, Christos Kamaras, Franziska Zell, Madelon Hossfeld, Olaf Groß, Achim Lother

**Affiliations:** 1 Institute of Experimental and Clinical Pharmacology and Toxicology, Faculty of Medicine, University of Freiburg, Albertstr. 25, 79104 Freiburg, Germany; 2 Institute of Neuropathology, Faculty of Medicine, Medical Center - University of Freiburg, Freiburg, Germany; 3 Division of Molecular Hematology, Department of Medicine I, Faculty of Medicine, Medical Center - University of Freiburg, Freiburg, Germany; 4 Signalling Research Centres BIOSS and CIBSS, University of Freiburg, Freiburg, Germany; 5 Interdisciplinary Medical Intensive Care, Faculty of Medicine, Medical Center – University of Freiburg, University of Freiburg, Freiburg, Germany

**Keywords:** Heart failure, Endothelial cells, Angiogenesis, Gene expression, Cell cycle, Hypertrophy

## Abstract

Cardiac hypertrophy is a key mechanism that allows the heart to adapt to increased load, but in the long term is associated with a higher risk for heart failure, arrhythmia, and death. During hypertrophic growth, cardiac myocytes signal to endothelial cells via vascular endothelial growth factor (VEGF) to promote angiogenesis and maintain myocardial oxygen supply. Insufficient angiogenesis leads to a decline in capillary density and drives the progression from compensated cardiac hypertrophy to heart failure. Here, we studied the time course of endothelial cell gene expression during heart failure development and identified transcriptional regulators of cell proliferation and angiogenesis. We applied transverse aortic constriction (TAC) in mice and isolated cardiac endothelial cells for RNA sequencing after 6 h and 1, 3, 7, or 28 days to create an inventory of gene expression during the course of cardiac hypertrophy and failure. Echocardiography revealed that decompensated heart failure occurred between days 7 and 28 after TAC. At the same time, we observed a switch in endothelial cell gene expression with an upregulation of proliferation markers in the hypertrophy state but downregulation in decompensated heart failure. Of note, endothelial cell cycle arrest occurred despite strong VEGF signaling from cardiac myocytes, indicating VEGF resistance. To investigate how endothelial cell proliferation is transcriptionally regulated, we performed a weighted gene co-expression network analysis and identified a module of 180 cell cycle-related genes. We predicted transcription factor 19 (TCF19), ATPase family AAA domain containing 2 (ATAD2), and transcription factor Dp-1 (TFDP1) to be central regulators of this gene module. Knockdown of *TCF19* and *ATAD2* by siRNA in HUVECs led to a downregulation of the marker of proliferation *MKI67* and repressed cell proliferation, tube formation, and cell migration, confirming their regulatory function. In heart tissue biopsies from patients with aortic stenosis, *TCF19* and *ATAD2* abundance were positively correlated with endothelial cell proliferation. TCF19 or ATAD2 control the expression of a gene network involved in endothelial cell proliferation and angiogenesis. Downregulation of TCF19 and ATAD2 is associated with endothelial cell cycle arrest and an impaired angiogenic response to VEGF signaling that may promote the transition from compensated cardiac hypertrophy to heart failure.

**Supplementary Information:**

The online version contains supplementary material available at 10.1007/s00395-025-01139-4.

## Introduction

Chronic heart failure is associated with substantial morbidity and mortality and is one of the leading causes for hospitalization in the United States and Europe [50]. The course of heart failure often remains stable over a longer period of time during which the heart undergoes a remodeling process, including progressive cardiac hypertrophy [55]. Cardiac hypertrophy allows to temporarily preserve contractile function but, in the long term, is associated with an increased risk for heart failure, arrhythmia, and death [55].

The heart's contractile function depends on an adequate supply of oxygen and exchange of metabolites, which mainly takes place in the capillaries, where circulating blood cells, endothelial cells, and cardiac myocytes are in close proximity to each other [26]. As cardiac myocytes in the adult heart are largely unable to proliferate, pathological hypertrophy is achieved by growth of individual cardiac myocytes [33, 55]. This increase in cardiac myocyte size requires a proportional formation of new capillaries to maintain sufficient oxygen supply to the heart tissue [15, 45]. Rarefaction of capillary density leads to myocardial hypoxia, aggravates the remodeling process, and facilitates the disease progression [35, 45]. The transition from compensated hypertrophy to heart failure induced by transverse aortic constriction coincides temporally with a decline in capillary density, suggesting a causal relationship between these processes [56].

Under steady state conditions, the majority of endothelial cells are quiescent. In response to hypoxia cardiac myocytes produce hypoxia-inducible factor 1α (HIF1α) and vascular endothelial growth factor A (VEGF-A). VEGF-A is a key pro-angiogenic factor, activating endothelial cells by acting on VEGF receptor 2 [15, 40, 45]. Increased VEGF-A signaling leads to the formation of tip cells, a highly specialized, low-proliferating endothelial cell subtype that forms filopodia and guides a newly forming vessel sprout towards the angiocrine signal [11]. Tip cells are followed by stalk cells, which proliferate rapidly and promote sprout elongation and lumen formation [11]. Effective angiogenesis requires a balance between endothelial cell proliferation, sprouting, and migration that is tightly coordinated by a network of receptors and ligands, including Notch/DLL4, ephrins, CXCR4/CXCL12, and others [40].

Most of the currently available treatments for heart failure aim at limiting the adverse effects of the sympathetic and the renin–angiotensin–aldosterone systems. In experimental studies, inhibition of HIF1α/VEGF-A signaling impairs angiogenesis during chronic pressure overload, leading to heart failure [20, 49], while VEGF-A treatment improves angiogenesis [12]. Thus, a number of attempts have been made to externally increase VEGF levels and thereby enhance angiogenesis in patients with coronary artery disease and myocardial ischemia [21, 29, 51, 52], however, none of these has proceeded into clinical application. A more precise understanding of how angiogenesis is controlled in heart failure may lead to the development of new therapeutic strategies. The transcriptome of cardiac endothelial cells shows organotypic characteristics that are closely linked to their function [30]. In this study, we analyzed cardiac endothelial cell gene expression at the transition from cardiac hypertrophy to failure and identified gene regulatory networks involved in the process of angiogenesis.

## Materials and methods

### Animal experiments

Male C57BL/6N mice were housed in individually ventilated cages in a specific pathogen-free facility, with 12 h daylight/dark cycles at 22 °C. Animals were fed a laboratory standard chow and water ad libitum. The study conforms to the ‘Guide for the Care and Use of Laboratory Animals’ published by the US National Institutes of Health and was performed after approval by the responsible Committees on the Ethics of Animal Experiments at the University of Freiburg (Regierungspräsidium, Freiburg, Germany, permit number: G-20/146).

### Human tissue samples

Snap-frozen left ventricular tissue from patients with aortic stenosis undergoing cardiac surgery was provided by the Cardiovascular Biobank Freiburg (ethical approval reference 393/16, study approval reference EK Freiburg 82/20).

### Transverse aortic constriction

Mice aged 8–10 weeks were used in this study. Two days before the surgery, the mice were acclimated to sweet drinking water containing 0.3% glucose. Analgesia was administered 30 min before surgery, 5 h after surgery, and every 6 h for the following two days via subcutaneous injection of buprenorphine (0.1 mg/kg). Overnight, the mice were provided with drinking water containing 0.3% glucose and 0.3 mg/mL buprenorphine. For surgery, mice were anesthetized with a single intraperitoneal dose of ketamine (100 mg/kg) and xylazine (6 mg/kg) and placed in a supine position. After skin incision, the salivary glands were retracted, and a partial thoracotomy was performed by a longitudinal incision of the cranial sternum (2–3 mm). A microsurgical clip (internal diameter 0.305 mm, adjusted to a 32G needle) was applied to the transverse aorta between the left carotid artery and the brachiocephalic trunk using a micro clip applicator. The thorax was closed using sutures, and the mice were allowed to recover on a warming pad until fully awake. The sham procedure was performed identically, except for clip application.

### Echocardiography

Echocardiography was performed in anesthetized mice (2 vol% isoflurane in 1 L/min O_2_) 6 h and 1, 3, 7, and 28 days after TAC, using a Vevo 3100 system with an MX55D probe operating at 40 MHz. Motion and wall thickness, as well as left ventricular cavity dimensions, were evaluated in the parasternal long-axis B-mode view. Fractional shortening was assessed in the short-axis M-mode view and analyzed using Vevo Lab software.

### Isolation of cardiac cells and nuclei for RNA-analysis

Mice were anesthetized with 0.3 mg/g body weight of thiopental i.p. before hearts were excised and snap-frozen in liquid nitrogen. Cardiac endothelial cells and myocyte nuclei were isolated for RNA-seq analysis or qPCR using fluorescence-assisted cell sorting as previously described [8, 48]. For isolation of endothelial cells, left ventricular (LV) tissue was mechanically dissected and then incubated for 30 min at 37 °C in 5 ml HBSS (Gibco) containing 4 mg Collagenase B (Sigma), 2.2 mg Hyaluronidase from bovine testes (Sigma), and 10 mM CaCl_2_. The resulting suspension was dissociated through repetitive pipetting, filtered using a 100 µm cell strainer (BD cell strainer) and centrifuged at 1000 × g for 5 min at 4 °C. The supernatant was discarded and the cell pellets were resuspended in PBS containing 2 mM EDTA and 0.5% BSA. Cells were incubated with *Griffonia simplicifolia* lectin conjugated to fluorescein (1:200) for 30 min at 4 °C and underwent centrifugation at 1000 × g for 5 min at 4 °C. The supernatant was discarded, followed by resuspension of the cell pellet in PBS containing 2 mM EDTA and filtration over a 70 µm cell strainer (BD cell strainer). Nucleated cells were then labelled with the fluorescent dye Draq5 (Cell Signaling Technology). Fluorescence-assisted cell sorting was conducted using an S3 Cell Sorter (Bio-Rad).

For isolation of cardiac myocyte nuclei, mouse hearts were mechanically dissected using gentleMACS Dissociator (Miltenyi) in lysis buffer containing 5 mM CaCl, 3 mM MgAc, 2 mM EDTA, 0.5 mM EGTA, 10 mM Tris–HCl (pH 8), protease inhibitor (Roche cOmplete Protease Inhibitor Cocktail) and DTT. Homogenized tissue was mixed with lysis buffer containing 0.4% Triton X-100, filtered with 50 µm cell strainer (BD cell strainer), and centrifuged at 1000 × g for 5 min. The resulting pellet was resuspended in 0.2% Triton X-100 containing lysis buffer, layered on 1 mM Saccharose, 3 mM MgAc, and 10 mM Tris–HCl (pH 8) in non-ionic water and centrifuged at 1000 × g for 5 min. The supernatant was discarded, pellet containing nuclei resuspended in PBS with 1% BSA, 300 mM Glycin, and 0.1% Tween 20, then incubated for 30 min first with anti-PCM-1 antibody (HPA023374, Sigma) and again for 30 min with anti-rabbit antibody conjugated to Alexa488 (A11034, Invitrogen).

After incubation, the suspension underwent centrifugation at 1000 × g for 5 min, supernatant was discarded, pellet resuspended in PBS containing 1 mM EDTA, and filtered using a 30 µm cell strainer (BD cell strainer). Nuclei were then labeled with the fluorescent dye Draq7 (Cell signaling technology). Fluorescence-assisted cell sorting was conducted using an S3 Cell Sorter (Bio-Rad).

### RT-qPCR

For RNA isolation, cells were lysed using RLT Plus lysis buffer (Qiagen). RNA isolation procedures followed the manufacturer's protocol as specified in the AllPrep DNA/RNA Micro Kit (Qiagen). RT-qPCR was performed using Quantitect Reverse Transcription Kit PCR (Qiagen) according to the manufacturer’s instructions. RT-qPCR was performed utilizing SsoAdvanced Universal SYBR Green Supermix and Thermo Cycler (BioRad) using ribosomal protein S29 (*Rps29, RPS29*) as a reference gene. Primer sequences are provided in Supplemental Table S01. Each sample was assessed in triplicates.

### RNA-seq library preparation

For RNA-seq, sequencing libraries were generated using NuGEN Ovation SoLo Kit (Tecan). Size selection was performed using AMPure XP Beads (Beckman Coulter). The quality of the resulting libraries was assessed using the High Sensitivity Assay (Agilent) with Bioanalyzer (Agilent). Libraries were sequenced in paired-end mode using Illumina NovaSeq sequencers.

### Bioinformatics

The European Galaxy platform (http://usegalaxy.eu) [53] was used for bioinformatic analyses. After quality control using FastQC, low-quality reads and adapters were removed using TrimGalore!. Reads were aligned to the Mus musculus genome (mm9) using RNA-STAR [9], followed by removal of PCR duplicates by using RmDup. Transcript abundancy was determined using htseq-count [1]. pyGenomeTracks [28] was used for data visualization. DESeq2 (*q* < 0.05) was executed for differential gene expression analysis [34]. Enrichment of biological processes among differentially expressed genes was performed using Cytoscape with ClueGo [5]. To determine the endothelial cell secretome at each time point after TAC, we used a curated ligand–receptor interaction database [37]. Differentially expressed ligands were subjected to functional enrichment analysis of biological processes using ClueGO.

### Weighted gene co-expression network analysis

To identify clusters of functionally related genes, a weighted gene co-expression network analysis (WGCNA) was performed [57]. Normalized counts of protein-coding genes (*n* = 10.144) were used, including those with an expression level of at least more than 10 normalized counts in all samples in a minimum of one condition to minimize background noise. Generating an adjacency matrix, bicor was applied for correlation calculations, designated as a signed hybrid network, and a soft threshold (*β* = 6) was utilized to ensure a scale-free topology (R^2^ > 0.8). The resulting values were converted into a topological overlap matrix (TOM). Hierarchical clustering was employed to detect modules containing at least 30 genes (minModuleSize = 30). Module eigengenes were calculated, clustered based on correlation, and merged using a height cut of 0.25 to combine similar modules. By correlating module eigengenes with condition traits (traits), like the timepoint or intervention, gene clusters showing significant associations with these traits were identified. Hub genes, representing those with high intramodular connectivity, were identified based on their module membership values [24].

To explore the internal network architecture of the orange module, a topological overlap matrix (TOM) was calculated to assess the connection strength between genes. Based on TOM similarity, the 50 strongest interaction partners were identified for each of the three selected hub genes. The resulting subnetworks were visualized using Cytoscape (v3.7.1) to illustrate intramodular connectivity and network positioning.

### Culture of human umbilical vein endothelial cells

Human umbilical vein endothelial cells (HUVEC, Lonza) were cultured and expanded up to passage 7 at 37 °C and 5% CO_2_. Cells were grown in Endothelial Growth Medium-2 (EGM-2, Lonza) with supplements containing hydrocortisone, hFGF-B, VEGF, R3-IGF-1, ascorbic acid, hEGF, GA-1000, heparin, and 10% fetal calf serum (FCS). To assess dose-dependent effects of VEGF-A, HUVECs were seeded in 12-well plates and cultured for 72 h in EGM-2 medium without VEGF, supplemented with 1% FCS and recombinant human VEGF-A (0, 50, 200 ng/ml). Medium was refreshed after 36 h and cells were harvested at 72 h for RNA isolation.

### siRNA knock-down

Cells were maintained in 12-well plates (Corning) until they reached 80–90% confluence. After 2 h of preconditioning with EBM-2 supplemented with 1% FCS, cells were transfected using Lipofectamine RNAiMAX (Invitrogen) with siRNAs targeting *TCF19* (siRNA ID: s13900; Ambion), *ATAD2* (siRNA ID: s26394; Ambion), *TFDP1* (siRNA ID: s14026; Ambion), or control siRNA (silencer select negative control; Ambion) following the manufacturer’s protocol. After 5 h, cells were washed with PBS, and the medium was replaced with EGM-2 containing 30% FCS. Following 12 h, the medium was changed to EGM-2 with 1% FCS.

### Tube formation assay

For tube formation assays, HUVECs were transferred 72 h after transfection into EBM-2 containing 2% FCS and then seeded onto preincubated and solidified basal membrane extract (Cultrex BME, Bio-Techne) in 24-well plates (Corning) at 32.000 cells per well. Cells were stimulated with 20 ng/ml of VEGF-A (293-VE, R&D systems) and fixated with 4% PFA after 4.5 h of incubation at 37 °C with 5% CO_2_. One image per well was captured from the center and analyzed using ImageJ Angiogenesis Analyzer module [6].

### Scratch migration assay

To assess in vitro migration capacity, HUVECs were cultured in EGM-2 with 10% FCS until they reached 85–90% confluence. 72 h after transfection, a scratch was made in the center of the 12-well plate (Corning) using a 10 µl pipette tip. Detached cells were removed by washing with PBS and replacing the medium with EGM-2 containing 1% FCS. Three images (top, center, bottom) of the scratch were captured at 100-fold magnification at 0, 3, 6, 12, and 24 h after scratch. For analysis, the cell-free area in each image was measured in a blinded manner, and the mean area for the three images per well was calculated using ImageJ. Relative wound closure was determined by subtracting the area at each time point from the initial area at 0 h and dividing by the initial area.

### Proliferation

To evaluate the effects of gene knockdown on cell proliferation in HUVECs, cells were seeded in 6-well plates (Corning) at a density of 100,000 cells per well in EGM-2 with 10% FCS. Transfections were performed as described and one central image per well at 100-fold magnification was captured at 0 h, 24 h, 48 h, and 72 h post-transfection. Image analysis and cell counting were conducted using the RTrees machine learning tool within the QuPath software [2].

### Ki67 staining

For Ki67 protein expression analysis, HUVECs were plated in 96-well plates (Corning), transfected, and fixed 72 h post-transfection with 4% paraformaldehyde (PFA) for 10 min. After washing with PBS, cells were permeabilized with 0.1% Triton X-100 and blocked with 1% albumin fraction V. Cells were then incubated for 1 h with a Ki67 primary antibody (1:100; ab279653, Abcam). Alexa Fluor 568-conjugated anti-mouse secondary antibody (1:400; A21134, Invitrogen) was used, and nuclei were stained with DAPI (1:10,000; D1306, Invitrogen). For each well, 3–4 randomly selected fluorescence images were captured using a Thunder Imager fluorescence microscope (Leica Microsystems). The images were analyzed in QuPath by calculating the ratio of Ki67-positive nuclei to DAPI-positive nuclei.

### Immunohistochemistry

Paraffin-embedded mouse hearts were sectioned at the basal region (3 µm thickness) for the assessment of cardiomyocyte cross-sectional area and capillary density. Sections were stained with Wheat Germ Agglutinin (WGA, Texas Red™, W21405, Invitrogen), Griffonia simplicifolia Lectin I (GSL I, FITC, FL-1101, Vector Laboratories), and DAPI (D1306, Invitrogen). Imaging was performed on a PhenoImager Fusion microscope (Akoya Biosciences), and spectral unmixing was carried out using InForm software (Akoya Biosciences). Capillary density was quantified in QuPath using a Random Trees Pixel Classifier, whereas cardiomyocyte cross-sectional area was determined by manual annotation of 100 transversely cut myocytes with centrally located DAPI-positive nuclei.

To study endothelial cell proliferation, mouse left ventricular tissue and human left ventricular biopsies (Biobank samples) were fixed in 4% paraformaldehyde (PFA) in 1 × PBS at 4 °C overnight, dehydrated in a sucrose gradient, and embedded in Tissue-Tek OCT compound (4583, Sakura) Cryosections were prepared using a Leica CM1950 cryostat and stored frozen until use. For immunofluorescence staining, sections were air-dried, refixed in 4% PFA for 15 min, and subjected to antigen retrieval in citrate buffer for 10 min at 95 °C. Sections were permeabilized with 0.5% Triton X-100 in PBS for 15 min and blocked with 5% BSA and 1% normal goat serum in PBS for 1 h at room temperature. Primary antibodies were diluted in blocking solution and incubated overnight at 4 °C (50 µL per section). Antibodies included anti-mKi67 (1:100, ab279653, Abcam) for murine and human samples, anti-ATAD2 (1:100, HPA029424, Sigma Aldrich) and anti-TCF19 (1:100, ab155391, Abcam) for human samples. After three washes in 0.1% Triton X-100 in PBS (5, 10, and 15 min), sections were incubated for 75 min at room temperature with Alexa Fluor-conjugated secondary antibodies (anti-rabbit A21245; anti-mouse A11031; both 1:300, Invitrogen) and lectins. Lectin staining was performed using Griffonia simplicifolia Lectin I (FITC, FL-1101, Vector Laboratories) for mouse samples and Ulex europaeus agglutinin I (UEA I, Alexa Fluor™ 594 conjugate, L32476, Invitrogen), respectively, for human samples. Nuclei were counterstained with DAPI (1:1000, D1306, Invitrogen) for 5 min, and coverslips were mounted with Dako Fluorescence Mounting Medium (S3023, Agilent). Fluorescence imaging of cryosections was performed on a Zeiss confocal microscope. Image analysis was conducted using Fiji with semi-automated macro-scripting.

## Results

### Transition from cardiac hypertrophy to failure during chronic pressure overload

**Fig. 1:**
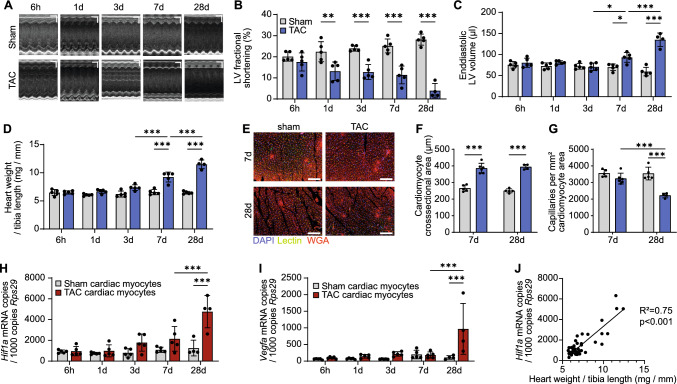
Left ventricular remodeling and function after pressure overload. C57BL/6 wildtype mice underwent transverse aortic constriction (TAC) for 6 h, 1 day, 3 days, 7 days, or 28 days. **A**, Representative echocardiography traces from left parasternal short-axis M-mode view. Bars 100 ms, 1 mm. **B-C**, Left ventricular fractional shortening (**B**) and end-diastolic volume (**C**) as determined by echocardiography. **D**, Heart weight to tibia length ratio after sham or TAC surgery. **E–F**, Mouse hearts were stained with wheat germ agglutinin, lectin, and DAPI (bars 100 µm) to determine cardiac myocyte cross-sectional area (**F**) and capillary density (**G**). **H-I**, Gene expression of Hypoxia inducible factor 1α (*Hif1a*, **H**) or Vascular endothelial growth factor A (*Vegfa*, **I**) in cardiac myocytes isolated from left ventricles after sham or TAC surgery. **J**, Pearson correlation of *Hif1a* expression and heart weight to tibia length ratio. *Rps29*, ribosomal protein S29. *n* = 4–5 per group. Means ± SEM. Two-way ANOVA with Bonferroni post hoc test. *P < 0.05; **P < 0.01; ***P < 0.001. For reasons of clarity, only statistical assessments between neighboring time points and respective control groups are shown

To assess the development of cardiac hypertrophy and failure, mice underwent TAC (*n* = 5 per group). Left ventricular function and morphology were assessed at defined time points after 6 h, 1 day, 3 days, 7 days, and 28 days of pressure overload (Fig. 1, Supplemental Table S02). Left ventricular contractility and inner diameter remained largely stable until day 7. On day 28, a loss of LV function and progressive left ventricular dilatation occurred (Fig. 1A-C). At the same time, lung weight to tibia ratio massively increased (d28 TAC 31.6 vs. sham 8.2 mg/mm; Supplemental Table S02) as a sign of decompensated heart failure. One animal died on day 21 and was excluded from further analysis.

Heart weight increased significantly within 7 days after TAC and further after 28 days (Fig. 1D, Supplemental Table S02). In line with this, histological analysis of left ventricular tissue revealed an increase in cardiomyocyte cross-sectional area at 7 and 28 days post-TAC (Fig. 1E-F). However, while capillary density was unchanged at 7 days, we observed a significant decline at 28 days after TAC (Fig. 1E, G). To determine gene expression changes in cardiac myocytes at the different time points, we isolated cardiac myocyte nuclei and performed RT-qPCR. At day 28 after TAC, we observed an increase in the expression of *Hif1a* and *Vegfa* (Fig. 1H, I). The increase in gene expression was well correlated with heart weight (*R*^2^ = 0.75, Fig. 1J), suggesting that the reduced capillary density in the hypertrophic heart at this time point causes cardiac myocytes hypoxia.

### Gene expression in cardiac endothelial cells after chronic pressure overload

**Fig. 2:**
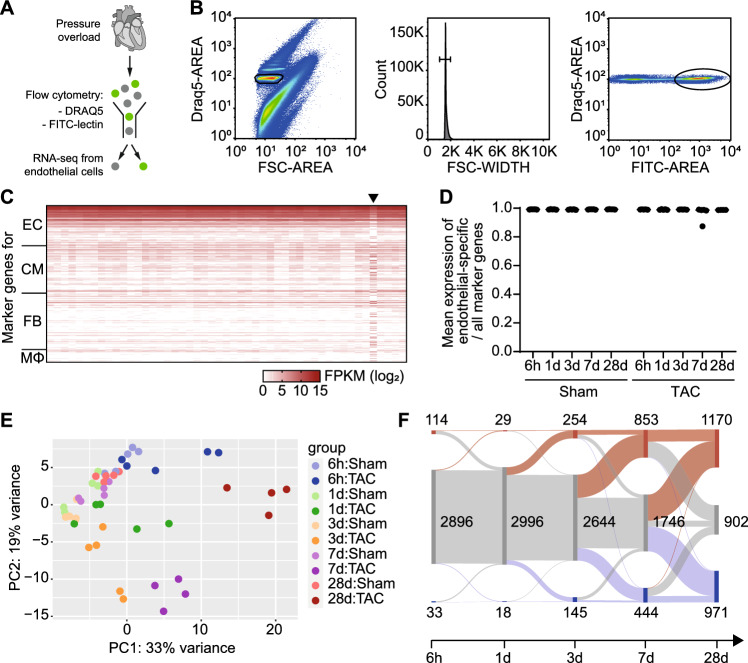
Isolation of left ventricular endothelial cells for RNA-seq. **A**, Endothelial cells from left ventricles were isolated by flow cytometry. **B**, Endothelial cells were identified using Draq5 as a nuclear stain and FITC-labelled lectin. **C**, Purity of endothelial cell fractions was assessed by determining the expression of previously identified [31] marker genes for endothelial cells, cardiac myocytes, fibroblasts, and macrophages. **D**, Ratio of the mean expression of endothelial cell marker genes compared to all marker genes in isolated cells. One sample was excluded due to contamination with non-endothelial cell RNA (C, triangle). **E**, Principal component analysis displaying heterogeneity of gene expression in endothelial cells after 6 h, 1 day, 3 days, 7 days, or 28 days of transverse aortic constriction (TAC). **F**, Numbers of differentially expressed genes (*q* < 0.05 vs. sham) during the time course after TAC. Displayed are genes that are differentially expressed at least at one time point. Red, upregulated; blue, downregulated; grey, not differentially expressed

**Fig. 3:**
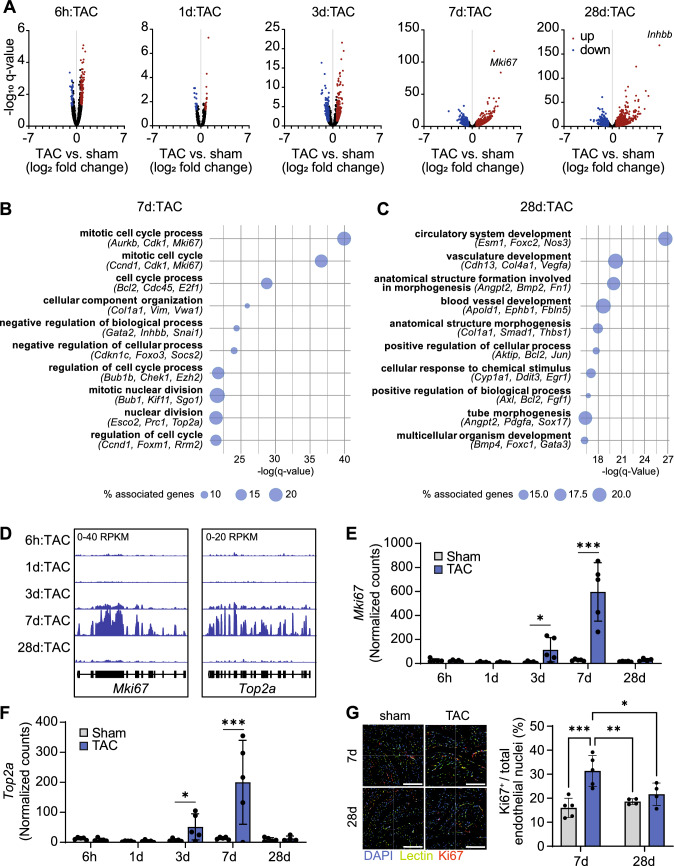
Gene expression in left ventricular endothelial cells at the transition from cardiac hypertrophy to heart failure. **A**, Volcano plots indicating differential gene expression at different time points after TAC. **B-C**, Biological processes enriched among genes that were differentially expressed at 7 days (**B**) or 28 days (**C**) after TAC as determined by Gene Ontology analysis (*P* < 0.05). **D-F**, Representative traces (**D**) and quantification (**E–F**) of RNA-seq data showing the expression of marker of proliferation Ki-67 (*Mki67*) and topoisomerase 2a (*Top2a*) in endothelial cells. *n* = 4–5 per group. DEseq2 with multiple comparison testing. *q < 0.05; ***q < 0.001 vs. respective sham. **G**, Abundance of MKI67 protein in cardiac endothelial cells at 7 days or 28 days after TAC was determined by immunohistochemistry (bars 100 µm). Means ± SEM. Two-way ANOVA with Bonferroni post hoc test. *P < 0.05; **P < 0.01; ***P < 0.001

To study gene expression in endothelial cells at the transition from compensated hypertrophy to failure, we isolated endothelial cells from LV tissue after TAC by flow cytometry (Fig. 2A-B, n = 4–5 per group). Purity of the obtained endothelial cell fractions was ensured by evaluating the expression of previously identified [31] marker genes for endothelial cells, cardiac myocytes, fibroblasts, and macrophages and calculating the ratio of mean endothelial marker gene expression to all marker genes (Fig. 2C-D, Supplemental Table S03). One sample (TAC d7) was excluded due to contamination with non-endothelial RNA. Principal component analysis indicated a shift in gene expression from day 1 until day 7, while gene expression at day 28 appeared distinct from all other time points (Fig. 2E). In total, we found 3,043 genes differentially expressed at any given time point after TAC (Supplemental Table S03). The number of differentially expressed genes increased over time. Interestingly, a large number of genes that were differentially expressed on day 7 returned to non-regulated on day 28, while other genes that had not been regulated before were differentially expressed at day 28 (Fig. 2F). On day 7, the majority of differentially expressed genes was associated with processes involved in cell cycling (Fig. 3A-B). In contrast, differentially expressed genes on day 28 were associated predominantly with vasculature development, including extracellular matrix components such collagens, integrins, and matrix metalloproteinases (Fig. 3C, Supplemental Table S04). A focused analysis of the endothelial cell secretome revealed a temporal progression from regulation of chemotaxis at early timepoints to extracellular matrix organization, angiogenesis, and signal transduction at days 3 and 7, followed by enrichment of cell migration and chemotaxis at day 28 (Supplemental Table S05).

We found several genes regulated at 28 days after TAC that have previously been described as regulators of angiogenesis, including protein tyrosine phosphatase 1B (*Ptpn1*, 1.5-fold up), cell cycle inhibitor p21 (cyclin-dependent kinase inhibitor 1A, *Cdkn1a*, 3.2-fold up), or sirtuin 1 (*Sirt1*, 1.4-fold down). Among the genes that were strongly upregulated at day 7 but returned to baseline expression on day 28 were marker genes of cell proliferation, such as *Mki67* or topoisomerase 2a (*Top2a*) (Fig. 3D-F). Immunohistochemical analysis of Ki67 protein expression confirmed these findings, indicating increased endothelial Ki67 abundance at 7 days after TAC, followed by a decline at 28 days (Fig. 3G). We concluded that the endothelial cell transcriptome undergoes a profound switch at the time of cardiac decompensation, with increased proliferation during the hypertrophic phase that returns to the quiescent state in the decompensated phase.

### Regulation of gene networks involved in endothelial cell cycle

**Fig. 4:**
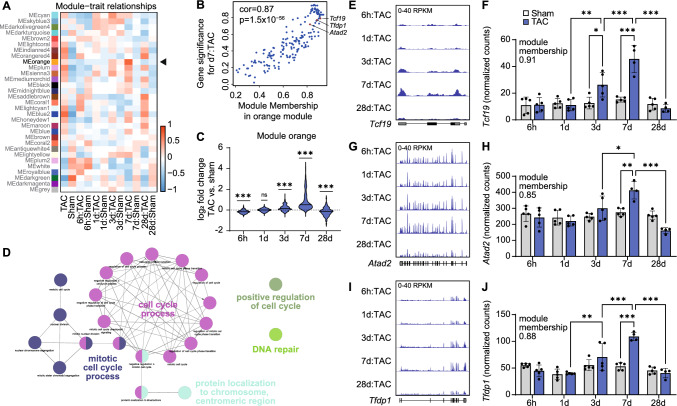
Weighted gene co-expression network analysis of cardiac endothelial cells. **A**, Module-trait relationship between gene modules and time points after transverse aortic constriction (TAC) or sham surgery. Arrow indicating module orange. **B**, Correlation of gene significance for day 7 after TAC versus membership in module orange. **C**, Distribution of changes in expression of genes from module orange between TAC and sham at different time points. *n* = 180 genes, one-sample t-test vs. hypothetical mean of zero. **D**, Biological processes enriched among genes from module orange as determined by Gene Ontology analysis (*P* < 0.05). **E-J**, Representative traces and quantification of RNA-seq data showing the expression of transcription factor 19 (*Tcf19*, **E–F**), ATPase family AAA domain containing 2 (*Atad2*, **G-H**), and transcription factor Dp-1 (*Tfdp1*, **I-J**) in endothelial cells. *n* = 4–5 per group. Means ± SEM. DEseq2 with multiple comparison testing. *q < 0.05; **q < 0.01; ***q < 0.001 vs. respective sham. For reasons of clarity, only statistical assessments between neighboring time points and respective control groups are shown

To understand the mechanisms of regulation involved in the transcriptomic switch at the time of decompensation, we performed WGCNA and identified 31 distinct gene modules (Fig. 4A, Supplemental Table S06). Module orange, containing 180 genes, showed a close relationship with d7 but not with d28 after TAC (Fig. 4A) and membership of genes to module orange closely correlated with their significance for d7 after TAC (Fig. 4B). In line with our previous findings, genes from module orange were upregulated on day 7 but downregulated at day 28 (Fig. 4C) and associated with cell cycle processes (Fig. 4D, Supplemental Table S07). We identified 3 hub genes of module orange as potential key transcriptional regulators of this gene module: transcription factor 19 (*Tcf19*), ATPase family AAA domain containing 2 (*Atad2*), and transcription factor Dp-1 (*Tfdp1*) were upregulated on day 7 after TAC when compared to respective sham but downregulated on day 28 when compared to day 7 (Fig. 4B, E-J). Their module membership score was 0.91, 0.85, or 0.88, respectively (Supplemental Table S06). The expression of *Tcf19*, *Atad2*, and *Tfdp1* showed a close correlation with each other and with the proliferation marker *Mki67* (Supplemental Figure S01).

### Impact of hub genes on angiogenesis in vitro

**Fig. 5:**
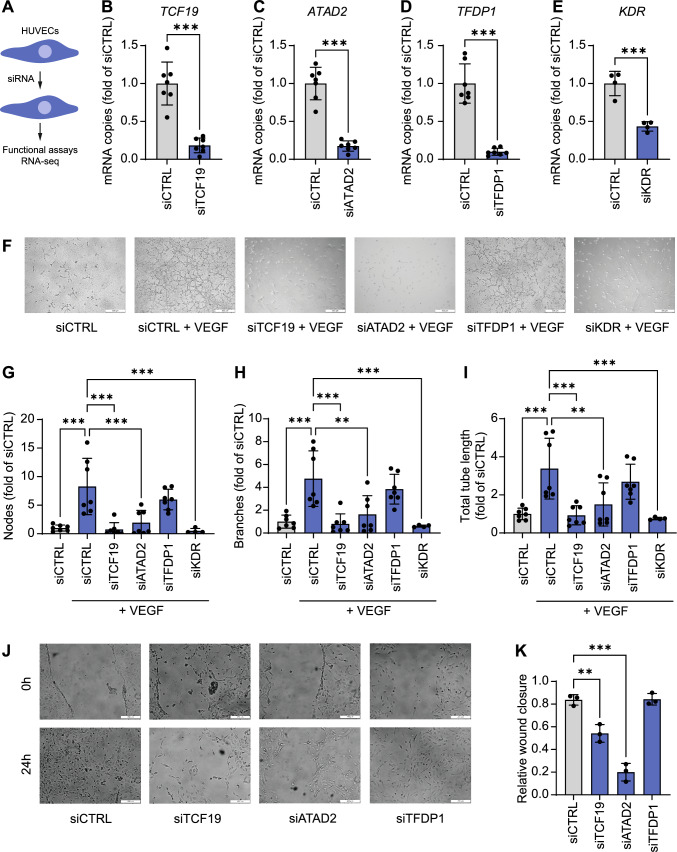
Impact of hub genes on angiogenesis in human endothelial cells. **A**, Human umbilical vein endothelial cells were transfected with siRNA against transcription factor 19 (*TCF19*), ATPase family AAA domain containing 2 (*ATAD2*), transcription factor Dp-1 (*TFDP1*), or VEGF receptor 2 (*KDR*). **B-E**, mRNA expression of *TCF19*, *ATAD2*, *TFDP1*, and *KDR* after siRNA transfection as assessed by RT-qPCR. *n* = 4–7 per group. Student’s t–test, ***P < 0.001 vs. siCTRL. **F-I**, Representative images of tube formation assay (F, bars 500 µm) and quantification of nodes (**G**), branches (**H**), and total tube length (**I**) in response to VEGF after siRNA transfection. *n* = 4–7 per group. **J**, Representative images of scratch-migration assay (**J**, bars 250 µm) and quantification of relative wound closure after siRNA transfection (**K**). *n* = 3 per group. One-way ANOVA with Bonferroni post hoc test. **P < 0.01; ***P < 0.001. Means ± SEM

To assess the functional role of the proposed transcriptional regulators in endothelial cells, we performed siRNA-mediated knockdown in HUVECs (Fig. 5A-E). After siRNA treatment, mRNA expression of *TCF19*, *ATAD2*, and *TFDP1* was reduced by 81.7%, 82.5%, or 90.1%, respectively. As a positive control, we performed knockdown of VEGF receptor 2 (*KDR*). Angiogenic potential of HUVECs in response to VEGF was assessed in tube formation assays (Fig. 5F-I). Number of nodes (Fig. 5G), number of branches (Fig. 5H), and the total tube length (Fig. 5I) were reduced after knockdown of *TCF19* or *ATAD2* similar to *KDR,* but not after knockdown of *TFDP1*. In scratch assays, wound closure after 24 h in HUVECs after knockdown of *TFDP1* was comparable to control but reduced after treatment with siRNA against *TCF19* or *ATAD2* (Fig. 5J-K). To test whether VEGF regulates *TCF19* and *ATAD2* expression, HUVECs were stimulated with increasing doses of VEGFA and mRNA levels were measured, however, no dose-dependent effect was observed (Supplemental Figure S02).

### Regulation of gene expression and cell cycle by TCF19, ATAD2, and TFDP1

**Fig. 6:**
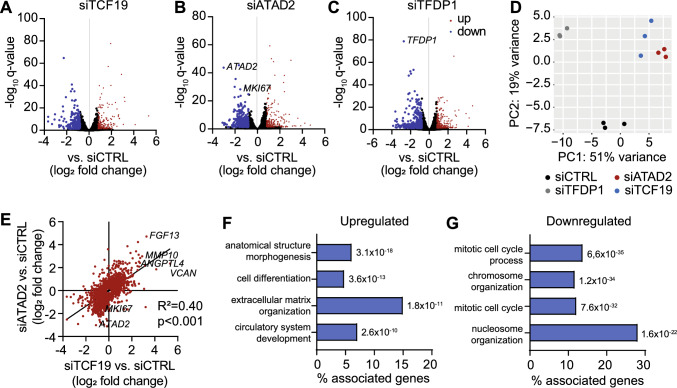
Hub genes in the regulation of endothelial cell gene expression. **A-C**, Differential gene expression (*q* < 0.05, *n* = 3 per group) in human umbilical vein endothelial cells (HUVECs) after transfection with siRNA against transcription factor 19 (*TCF19*, **A**), ATPase family AAA domain containing 2 (*ATAD2*, **B**), or transcription factor Dp-1 (*TFDP1*, **C**) as determined by RNA-seq. **D**, Principal component analysis displaying heterogeneity of gene expression after siRNA transfection. **E**, Pearson correlation of gene expression changes after transfection with siRNA against *TCF19* or *ATAD2*. **F-G**, Selected biological processes enriched among genes that were concordantly up- (**F**) or downregulated (**G**) after transfection with siRNA against *TCF19* or *ATAD2* as derived from Gene Ontology

To confirm the regulatory function of the predicted hub genes in endothelial cell gene expression and cell cycling, we performed RNA-seq from HUVECs. After siRNA-mediated knockdown of *TCF19*, *ATAD2*, and *TFDP1* we found 503 genes, 520 genes, or 608 genes differentially expressed (*q* < 0.05, |log2 fold change|> 0.8; Fig. 6A-C, Supplemental Table S08). Notably, principal component analysis indicated a close proximity of gene expression after knock-down of *TCF19* and *ATAD2* (Fig. 6D), which was further supported by a strong correlation of their expression profiles (Fig. 6E). Out of 175 human orthologue genes in module orange, 88 genes were differentially expressed after knock-down of one of the candidate genes with an overlap of 39 genes between *TCF19* and *ATAD2* knock-down (Supplemental Figure S03)*.* Genes that were concordantly downregulated in both groups were associated with mitotic cell cycle, while upregulated genes were predominantly linked to extracellular matrix organization (Fig. 6F-G, Supplemental Table S09), similar to what we had observed in primary cardiac endothelial cells on day 7 and 28 after TAC (Fig. 3B-C, Supplemental Table S04).

**Fig. 7:**
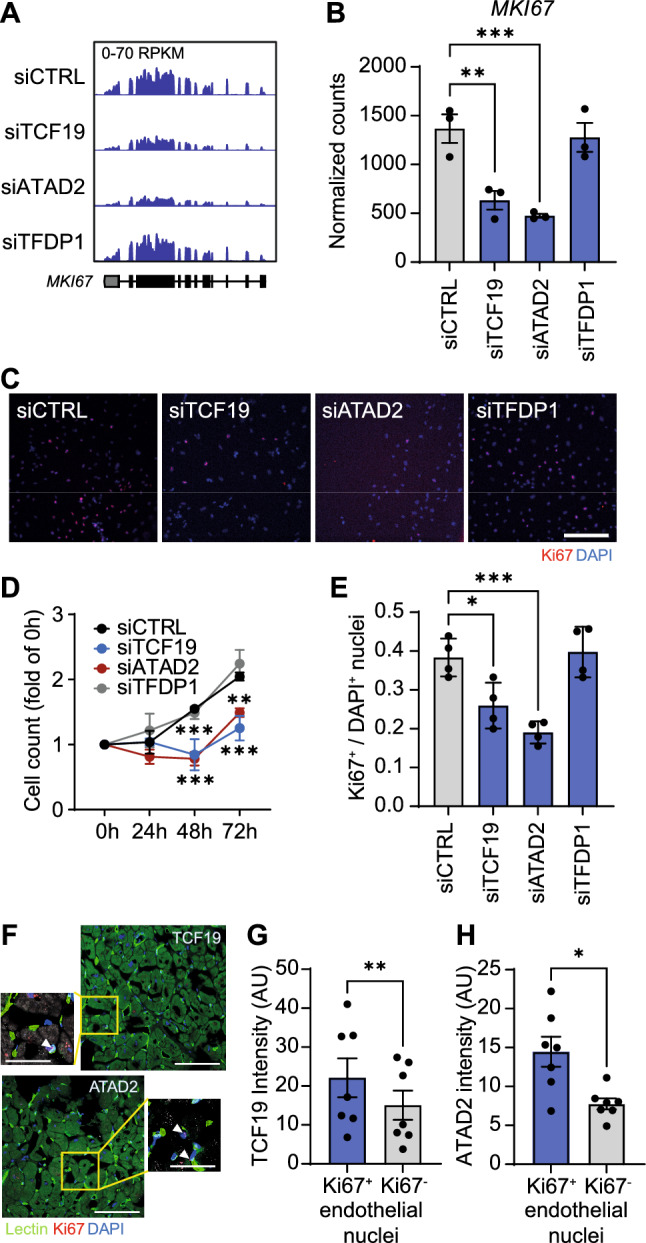
Impact of hub genes on human endothelial cell proliferation. **A-B**, Representative traces (**A**) and quantification (**B**) of RNA-seq data showing the expression of the marker of proliferation Ki-67 (*MKI67*) in human umbilical vein endothelial cells after siRNA transfection. *n* = 3 per group. DEseq2 with multiple comparison testing. **q < 0.01; ***q < 0.001. **C**, HUVECs were transfected with siRNA, treated with VEGF for 72 h, and stained with DAPI (blue) and an anti-Ki67 antibody (red). Bars 200 µm. **D**, Cell count of HUVECs at 0, 24, 48, and 72 h of VEGF treatment. Two-way ANOVA with Bonferroni post hoc test. **P < 0.01; ***P < 0.001. **E**, Ratio of Ki67^+^ to DAPI^+^ nuclei after 72 h. One-way ANOVA with Bonferroni post hoc test. *P < 0.05; ***P < 0.001. **F–H**, Abundance of TCF19 and ATAD2 in nuclei of Ki67^+^ proliferating versus Ki67^−^ non-proliferating endothelial cells in heart tissue biopsies from patients with aortic stenosis was determined by immunohistochemistry (*n* = 7 per group, bars 100 µm, bars in insert 25 µm). Paired t-test. *P < 0.05; **P < 0.01. Means ± SEM

Marker of proliferation *MKI67* was significantly downregulated after knockdown of *TCF19* or *ATAD2* but not of *TFDP1* (Fig. 7A-B, Supplemental Table S08). In line with this, proliferation rate and the number of Ki67^+^ HUVECs was lower after treatment with siRNA against *TCF19* or *ATAD2* but not *TFDP1* when compared to control (Fig. 7C-E). To validate these findings in human heart tissue, we assessed the abundance of ATAD2 and TCF19 in left ventricular biopsies from patients with aortic stenosis (*n* = 7, Supplemental Table S010). We observed a higher expression of TCF19 and ATAD2 in Ki67^+^ proliferating endothelial cells compared to Ki67^−^ endothelial cells (Fig. 7F-H). Thus, we conclude that TCF19 and ATAD2 control a gene expression network involved in endothelial cell proliferation and angiogenesis and their downregulation is linked to the cell cycle arrest observed at the transition to heart failure.

## Discussion

In this study, we created an inventory of endothelial cell gene expression during the time course of left ventricular pressure overload. We observed a downregulation of a cluster of cell cycle-related genes at the transition from cardiac hypertrophy to failure, indicating endothelial cell cycle exit despite high levels of VEGF. We identified TCF19 and ATAD2 as key regulators of this gene network in vivo*,* assessed their functional role in vitro, and confirmed their association with endothelial cell proliferation in human heart tissue. We conclude that TCF19 and ATAD2 are key regulators of endothelial cell proliferation and their downregulation inhibits angiogenesis downstream of VEGF signaling.

A dense capillary network is required to maintain contractile function and prevent the onset of heart failure in cardiac hypertrophy [16, 20, 49]. In our study, decompensation of left ventricular function occurred between 7 and 28 days after transverse aortic constriction. At the same time, we observed a decline in capillary density and a turning point in endothelial cell gene expression. Numerous genes that have been strongly regulated by day 7 showed inverse regulation on day 28, many of them related to the mitotic cell cycle. We conclude that endothelial cells undergo cell cycle block at the time of decompensation. This is in line with two previous reports describing a transient upregulation of cell cycle genes in heart tissue [14] or isolated endothelial cells [13] after one week followed by a decline after 8 weeks of pressure overload. Lineage-tracing experiments revealed that vascularization of the border zone myocardium after ischemia depends on clonal expansion of resident endothelial cells rather than circulating stem cells [23, 25]. It is, therefore, plausible that a lack of endothelial proliferation capacity impairs angiogenesis and causes heart failure.

It is noteworthy that endothelial cells stop proliferating despite high levels of VEGF-A and HIF1α expression in cardiac myocytes at day 28. We hypothesize that during the time course after TAC endothelial cells may become less responsive to VEGF signals, leading to impaired angiogenesis, which further aggravates cardiac myocyte hypoxia and subsequent VEGF expression in cardiomyocytes. This implies that the therapeutic administration of VEGF to improve angiogenesis—in a situation in which endogenous VEGF levels are already elevated—would not provide benefit. Results from clinical trials are ambiguous. While in one study, VEGF gene transfer improved exercise capacity in patients with coronary artery disease [51], this could not be confirmed in other trials [21, 52]. Myocardial perfusion as assessed by single-photon emission computed tomography was similar after VEGF gene transfer or placebo treatment [21, 52].

We have already shown that activation of the mineralocorticoid receptor, as seen in chronic heart failure, suppresses the expression of VEGF receptor 2 and counteracts VEGF signaling in endothelial cells [32]. In the present study, we observed a 17% downregulation of VEGF receptor 2 at day 28. However, it seems questionable whether this relatively moderate change alone limits angiogenesis capacity. Thus, the lack of response to VEGF may be related to changes downstream of VEGF receptor 2 signaling in endothelial cells. We found several genes regulated in cardiac endothelial cells that have previously been associated with cardiac angiogenesis. *Sirt1*, for example, a gene that was downregulated at day 28 after TAC, promotes endothelial cell proliferation [58] and is involved in sprouting angiogenesis [47]. Knock-out of *Sirt1* in mice leads to reduced capillary density in the heart [47]. Deletion of *Ptpn1*, a gene we found upregulated after TAC, protects from pressure overload- [16] or aging-related [4] left ventricular dysfunction. In both models, *Ptpn1* deletion improved capillary density [4, 16].

To further explore the mechanisms that control cell proliferation in endothelial cells from pressure overloaded hearts, we performed a gene regulation network analysis and identified TCF19 and ATAD2 to be upstream regulators of a cluster of cell cycle-related genes. To increase the robustness of our findings, we assessed publicly available datasets [13, 14] and could confirm an upregulation of *Tcf19* and *Atad2* in heart tissue or isolated endothelial cells, respectively, at 7 but not at 21 or 56 days after TAC. In contrast, no significant regulation could be found after experimental myocardial infarction [3], indicating that the regulation of *Tcf19* and *Atad2* may be specific for heart failure with more pronounced cardiac myocyte hypertrophy. ATAD2 is a chromatin-binding protein and transcriptional co-activator that belongs to the AAA protein family [7, 39]. ATAD2 was found to be associated with active chromatin regions and factors involved in DNA replication, DNA repair, and mitosis [39]. Upregulation of ATAD2 has been observed in various solid malignancies, including prostate, ovarial, and colorectal cancer, and was associated with chemoresistant phenotypes [41]. We found it particularly interesting that in stomach cancer cells, ATAD2 is upregulated during hypoxia in a Hif1α-dependent manner and promotes cell proliferation [42]. In addition, downregulation of ATAD2 in colon cancer cell lines suppressed VEGF secretion [19]. Transcription factor TCF19 is known as a regulator of pancreatic β cell mass and as a risk locus for type 1 diabetes [22]. Knockdown of TCF19 in vitro reduces β cell proliferation [22] and inhibits insulin secretion [43]. In hepatocellular carcinoma cells, TCF19 interacts with p53 and increases cell proliferation as well as the expression of metabolism-related genes [38]. Recently, TCF19 has been proposed as a driver of cell proliferation in lung cancer [54]. The role of ATAD2 and TCF19 in cardiovascular disease was so far unknown. In the heart, ATAD2 and TCF19 expression can be detected in all major cell populations, including a subset of endothelial cells [27]. We show here that knockdown of *TCF19* or *ATAD2* in HUVECs disturbed proliferation, migration, and tube formation, indicating a regulatory function in angiogenesis.

While VEGF is usually considered a pro-angiogenic factor, one may speculate that endothelial cell cycle block may rather be a consequence than cause of high VEGF expression after TAC. It has been shown that very high levels of VEGF block endothelial cell proliferation and cause angiogenesis arrest [46]. Excessive cell proliferation—as in cancer cells—may induce replication stress and cause DNA damage, and accordingly cell cycle activity is controlled by several checkpoints [36]. VEGF regulates the expression of cell cycle inhibitor p21 (cyclin-dependent kinase inhibitor 1A, *CDKN1A*), resulting in an inverted U-shape with low proliferation of endothelial cells at low and very high VEGF levels and high proliferation at medium VEGF levels [46]. Vice versa, endothelial cell-specific deletion of the tumor-suppressor p53 enhanced proliferation, improved capillary density, and prevented adverse cardiac remodeling after pressure overload [17]. We found p21 upregulated at day 28 after TAC and after siRNA knockdown of TCF19. The downregulation of TCF19 and ATAD2 observed here, together with an upregulation of p21, could represent such a protective mechanism. However, neither overexpression of Hif1α [10] nor treatment with increasing concentrations of VEGF altered the expression of *TCF19* or *ATAD2* in HUVECs, which argues against this hypothesis and suggests that other factors, yet to be identified, are involved in their regulation in endothelial cells.

### Perspectives and limitations

In conclusion, we found an association of TCF19 and ATAD2 expression with endothelial cell proliferation during cardiac hypertrophy in mice and humans. Our data indicate that TCF19 and ATAD2 act as transcriptional regulators of angiogenesis and may serve as new therapeutic targets beyond the classical VEGF signaling pathway. From a translational point of view, therapeutic restoration of TCF19 and ATAD2 expression could improve the angiogenic capacity of endothelial cells in the heart and provide benefit for patients with heart failure. However, gain-of-function of TCF19 and ATAD2 is associated with cell proliferation in cancer [41, 54]. Therefore, potential interactions between cardiovascular disease and cancer need to be carefully considered [44]. Further studies are needed to identify signaling cascades that lead to the downregulation of TCF19 and ATAD2 in heart failure. Finally, coronary blood flow in heart failure is not only disturbed by reduced capillary density but also by altered metabolic and neurohumoral signaling, impaired vasodilation and vasoconstriction, and increased extravascular compression [18], suggesting that complementary strategies are needed to improve heart failure-related outcomes [26].

## Supplementary Information

Below is the link to the electronic supplementary material.

Supplementary file1 (XLSX 29 KB)

Supplementary file2 (XLSX 8682 KB)

Supplementary file3 (XLSX 172 KB)

Supplementary file4 (DOCX 456 KB)

Supplementary file5 (XLSX 10 KB)

Supplementary file6 (XLSX 12 KB)

Supplementary file7 (XLSX 18993 KB)

Supplementary file8 (XLSX 1226 KB)

Supplementary file9 (XLSX 130 KB)

Supplementary file10 (XLSX 9357 KB)

## Data Availability

Raw data are accessible via BioProject IDs PRJNA1190829 and PRJNA1190831.
