# Supplementary material for: Downregulation of TCF19 and ATAD2 causes endothelial cell cycle arrest at the transition from cardiac hypertrophy to heart failure
Source: Basic Res Cardiol. 2025 Sep 17;120(6):1209–24. doi: 10.1007/s00395-025-01139-4 (PMC12680863; doi:10.1007/s00395-025-01139-4)
Supplement: Supplementary file 4 — Supplementary file4 (DOCX 456 KB) [file 395_2025_1139_MOESM4_ESM.docx]

**Supplemental table S01: Primer sequences**

| **Gene** | **sequence [5'--3']** | **species** |
| --- | --- | --- |
| ATAD2_sense | AGT CAT CTG AAG AGG GTG AAG A | human |
| ATAD2_antisense | CGC TTC TGA TTC TCT TCT TCT CC | human |
| TCF19_sense | ATC TCT ACA CCT TCC ACC CC | human |
| TCF19_antisense | CTT GGC TGC TGT GGT CTT C | human |
| TFDP1_sense | CCA CGA TGA CAT AGA AGT GCT G | human |
| TFDP1_antisense | TGT CAC GTA TGG CTC CAG AG | human |
| RPS29_sense | GGT TCT CGC TCT TGT CGT GTC | human |
| RPS29_antisense | ATA TCC TTC GCG TAC TGA CGG | human |
| VEGFR2_sense | GAA CAT TTG GGA AAT CTC TTG C | human |
| VEGFR2_antisense | CGG GAA GAA CAA TGT AGT CTT TGC | human |
| Hif1a_sense | TCT GGA TGC CGG TGG TCT AGA CAG T | mouse |
| Hif1a_antisense | GTT AGC ACC ATC ACA AAG CCA TCT AG | mouse |
| Rps29_sense | ATG GGT CAC CAG CAG CTC TA | mouse |
| Rps29_antisense | AGC CTA TGT CCT TCG CGT ACT | mouse |
| Vegfa_sense | AGA GCA ACA TCA CCA TGC AG | mouse |
| Vegfa_antisense | CGC CTT GGC TTG TCA CAT | mouse |

**Supplemental table S10: Patient characteristics**

| Male / female sex (n) | 5 / 4 | n = 9 |
| --- | --- | --- |
| Age (years, mean ± SEM) | 60.6 ± 4.9 | n = 9 |
| Ejection fraction (%, mean ± SEM) | 51.3 ± 5.3 | n = 8 |
| Coronary artery disease (%) | 55.6 | n = 9 |
| Hypertension (%) | 55.6 | n = 9 |
| Kidney disease (%) | 11.1 | n = 9 |
| Obesity (%) | 44.4 | n = 9 |

**Supplemental Figure S01:**

**
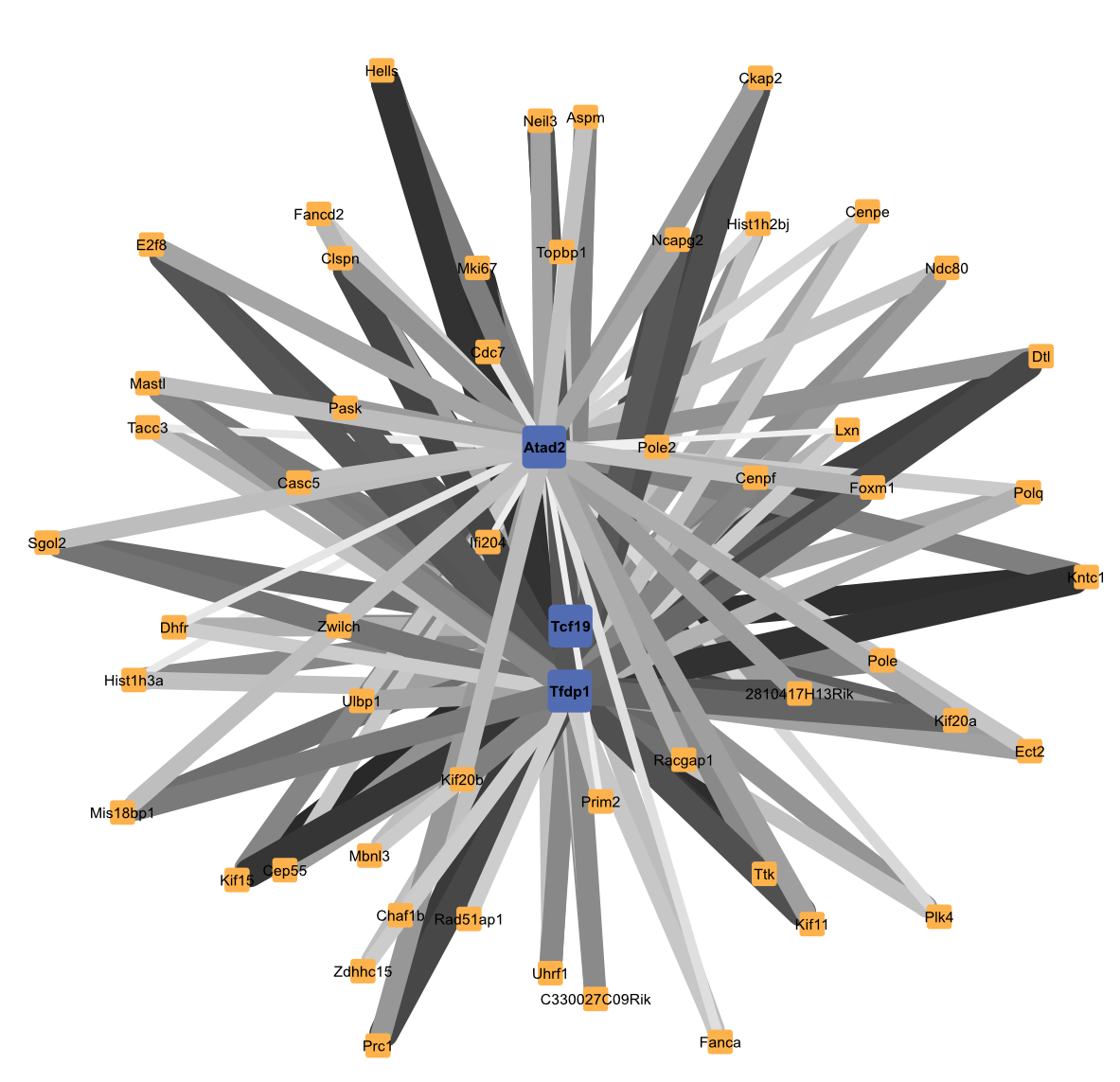
**

**Network regulation by hub genes in module orange.** TOM-based subnetworks of the top 50 interaction partners of *Tcf19*, *Atad2* and *Tfdp1* within the orange module. Edge thickness and color intensity represent the strength of topological overlap between genes.

**Supplemental Figure S02:**


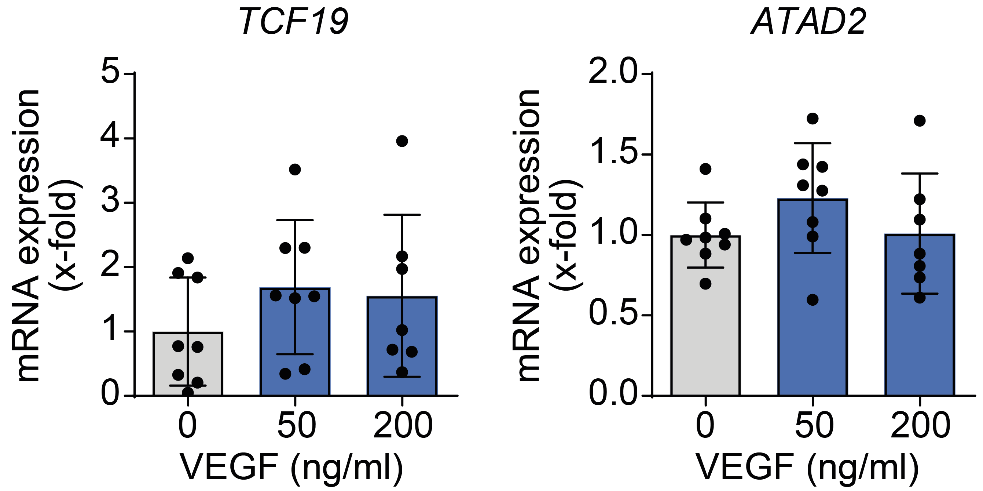


**VEGF-dependent gene expression in HUVECs.** mRNA expression of *TCF19* and *ATAD2* in HUVECs 72 hours after treatment with VEGF was assessed by qRT-PCR.

**Supplemental Figure S03:**

**
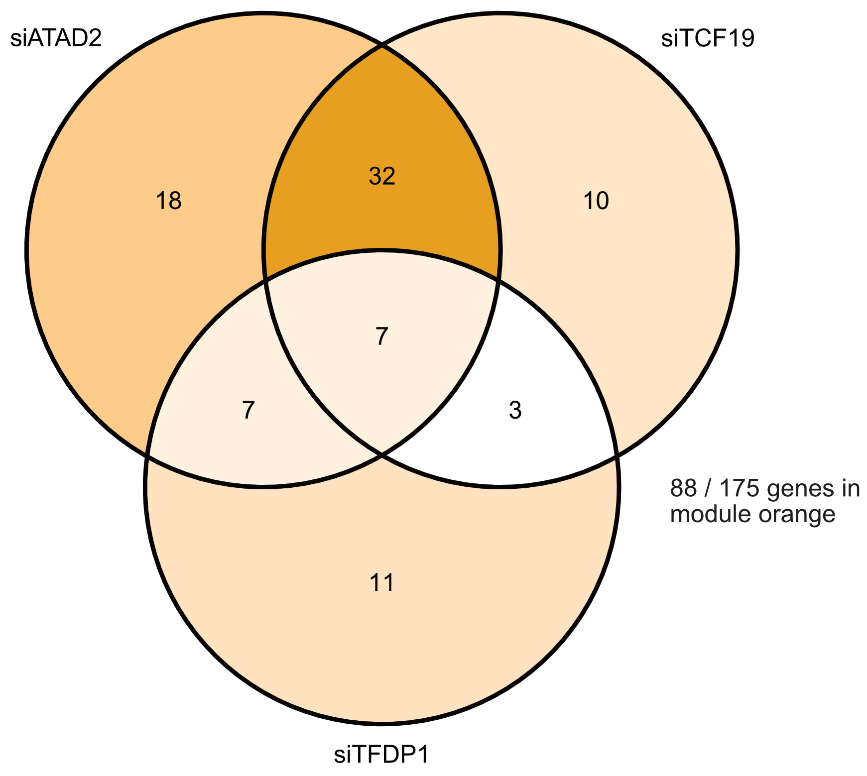
**

**Gene expression after knock-down of hub genes.** Venn diagram showing genes within module orange that were differentially expressed after siRNA knockdown of *ATAD2*, *TCF19*, or *TFDP1*.
